# Building an interdisciplinary pain medicine and palliative care program in Ethiopia

**DOI:** 10.7189/jogh.10.010317

**Published:** 2020-06

**Authors:** Salahadin Abdi, Ethan Dmitrovsky

**Affiliations:** 1 Department of Pain Medicine, The University of Texas MD Anderson Cancer Center, Houston, Texas, USA; 2 Frederick National Laboratory for Cancer Research, Frederick, MD, USA

While America confronts a crisis in the misuse and overuse of opiates, Africa has the opposite problem. Opiates are essentially unavailable in Ethopia (other than in a few urban hospitals), leaving those in chronic pain – including cancer patients – to suffer without relief. And this predicament is worsening.

Cancer is the second-leading cause of death worldwide [1]. But as cancer mortality continues to decline [2] in the United States, the disease is gaining ground against infectious disease [3] as a major cause of mortality in low-to-middle-income countries, like those in sub-Saharan Africa. The root causes for this unexpected increase include the growing and aging populations [4] along with changes in lifestyle in these countries.

With cancer comes pain, yet more than 3.2 billion people worldwide lack access to morphine for adequate pain relief [5]. Indeed, the World Health Organization recommends opiates for moderate to severe cancer pain [6]. It placed this analgesic on its list of essential medicines [7]. Sadly, access to this drug class falls far short of the clinical need. About four in five patients with terminal cancer experience moderate to severe pain from lack of adequate pain control [4].

In Ethiopia, cancer is an especially heartbreaking condition because Ethiopia has scarce funds to invest in the latest cancer treatments [1], let alone prevent or treat cancer pain that burdens those same patients. If patients reach a hospital and are diagnosed with cancer, the disease is usually quite advanced and patients are in unbearable pain. Cancer specialists are torn over how best to act.

Cancer doctors are taught to ease pain in cancer sufferers wherever they can. This dictum likely drove the Montreal declaration [6] by the International Association for the Study of Pain, which proclaimed that access to pain management is a basic human right. But a proclamation alone will not produce results unless it is tied to a strategy that drives change.

## CANCER GAINING ON AIDS AS A HEALTH THREAT

Ethiopia is where AIDS and other communicable diseases exact a heavy toll. However, progress against these maladies has been made with improved treatment and prevention [3]. As these illnesses become better controlled, the incidence of non-communicable diseases like cancer will consequently rise.

Cancer is already the third most common cause of death in sub-Saharan Africa [8]. There is therefore a priority to improve pain management and palliative care in these countries. Ethiopia is the second most populous nation in Africa, with nearly 100 million citizens [9]. Its cancer burden casts a shadow over the public’s health in sub-Saharan Africa. In 2016 alone, about 60 000 new cancer cases were recorded and, unfortunately, 44 000 people succumbed to this illness [3]. This statistic likely underestimates the problem because many cancer cases are not tracked, let alone treated.

## TAKING STEPS TO TREAT CANCER AND PAIN

Cancer treatment has become a health priority in Ethiopia, and with it, a push to address the accompanying pain. The government is building five treatment centers and expanding the existing center in Addis Ababa [10]. The growing infrastructure will offer clinical services not yet available in Ethiopia (except at Black Lion Hospital), such as radiation oncology. With help from the American Cancer Society’s “Treat the Pain” initiative, cancer clinicians in African countries can acquire pain medicine for their patients, including opioid analgesics [5].

Even if these medicines become available, there must be a clinical workforce that knows how to use them. Many physicians in Ethiopia are reluctant to prescribe what opioid medication they have, leaving it to expire unused. Accordingly, the Federal Ministry of Health of Ethiopia seeks to develop a workforce versed in different areas of cancer care, including pain management and palliative care.

To begin to respond to this urgent need, a pain medicine - palliative care and hospice training pilot program was launched by Dr Salahadin Abdi at the Ayder Specialty Hospital in Mekelle. The first class of seven physicians from several medical disciplines was recruited, and a second class will matriculate soon in Bahir Dar, Addis Ababa and possibly Gondar.

**Figure:**
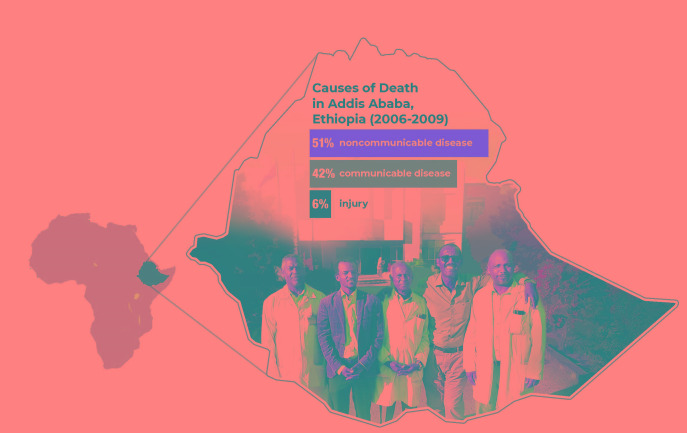
Photo: Some of the Ethiopian physician participants in the pain management education program. The data shown represents causes of death in Addis Ababa, Ethiopia for the most recently recorded timeframe (Prev Chronic Dis, 2019, 9:E84), revealing the high incidence of noncommunicable diseases (such as cancer) in the region (from the collection of Joseph Meyer, used with permission). The physicians shown above have permitted the use of their likenesses in this publication.

This two-year clinical fellowship is a demonstration project. It will produce Ethiopian physicians who are experts in pain medicine as well as palliative and hospice care. They will serve patients at their respective centers and simultaneously educate the next generation of medical professionals who could be deployed elsewhere in Ethiopia. Trained in all types of pain management, the fellows are also cautioned to establish barriers to avoid the opioid abuse crisis now engulfing developed countries.

Time will tell if this training initiative is successful or sustainable. But it is a good place to start. It plants the seeds of a potential solution that could be duplicated elsewhere. Ultimately, this could become a vital tool to combat a looming calamity, now hiding in plain sight, in global cancer care.
